# Clock gene *Per2* as a controller of liver carcinogenesis

**DOI:** 10.18632/oncotarget.11037

**Published:** 2016-08-03

**Authors:** Ali Mteyrek, Elisabeth Filipski, Catherine Guettier, Alper Okyar, Francis Lévi

**Affiliations:** ^1^ INSERM and Paris Sud University, UMRS 995, Team « Cancer Chronotherapy and Postoperative Liver », Campus CNRS, Villejuif F-94807, France; ^2^ Assistance Publique-Hopitaux de Paris, Department of Medical Oncology and Laboratory of Anatomy and Pathologic Cytology, Hôpital Paul Brousse, Villejuif F-94800, France; ^3^ Istanbul University Faculty of Pharmacy, Department of Pharmacology, Beyazit TR-34116, Istanbul, Turkey; ^4^ Warwick Medical School, Cancer Chronotherapy Unit, Coventry, CV4 7AL, United Kingdom

**Keywords:** Circadian rhythm, Per2 gene, Hepatocellular carcinoma, Molecular clock, Cell cycle genes

## Abstract

Environmental disruption of molecular clocks promoted liver carcinogenesis and accelerated cancer progression in rodents. We investigated the specific role of clock gene Period 2 (Per2) for liver carcinogenesis and clock-controlled cellular proliferation, genomic instability and inflammation. We assessed liver histopathology, and determined molecular and physiology circadian patterns in mice on chronic diethylnitrosamine (DEN) exposure according to constitutive Per2 mutation. First, we found that Per2^m/m^ liver displayed profound alterations in proliferation gene expression, including c-Myc derepression, phase-advanced Wee1, and arrhythmic Ccnb1 and K-ras mRNA expressions, as well as deregulated inflammation, through arrhythmic liver IL-6 protein concentration, in the absence of any DEN exposure. These changes could then make Per2^m/m^ mice more prone to subsequently develop liver cancers on DEN. Indeed, primary liver cancers were nearly fourfold as frequent in Per2^m/m^ mice as compared to wild-type (WT), 4 months after DEN exposure. The liver molecular clock was severely disrupted throughout the whole carcinogenesis process, including the initiation stage, i.e. within the initial 17 days on DEN. Per2^m/m^ further exhibited increased c-Myc and Ccnb1 mean 24h expressions, lack of P53 response, and arrhythmic ATM, Wee1 and Ccnb1 expressions. DEN-induced tumor related inflammation was further promoted through increased protein concentrations of liver IL-6 and TNF-α as compared to WT during carcinogenesis initiation. Per2 mutation severely deregulated liver gene or protein expressions related to three cancer hallmarks, including uncontrolled proliferation, genomic instability, and tumor promoting inflammation, and accelerated liver carcinogenesis several-fold. Clock gene Per2 acted here as a liver tumor suppressor from initiation to progression.

## INTRODUCTION

Hepatocellular carcinoma (HCC) is the sixth most frequent cancer and the third most common cause of cancer mortality worldwide [1]. Clock gene *Per2* plays a central role in the prominent circadian regulation of liver metabolism and proliferation [2, 3]. Here we aimed at determining the role of *Per2* in liver carcinogenesis. To address this question, chronic exposure to diethylnitrosamine (DEN) was used as a model for inducing rodent HCC, whose histology and genetic signature have been considered to be similar to poor prognosis human HCC [4].

Circadian rhythms with an about 24-h period characterize the expression patterns of 10-40% of mouse liver transcripts [5, 6]. These molecular rhythms are controlled by a genetic molecular clock in each cell [7]. The molecular oscillator involves an activation loop, where *Clock* or *NPas2* and *Bmal1* activate the transcription of *Period (Per)* genes 1, 2 and 3, *Cryptochrome (Cry)* genes 1/2, *Rev-erbα/β* and *Dec 1/2*. In turn, the *Per, Cry, Rev-erb and Dec* genes inhibit their own transcription and that of other clock genes through the interaction of their protein heterodimers with the CLOCK::BMAL1 protein heterodimer or clock gene promoters [8]. Circadian clocks regulate nutrient and xenobiotic metabolism, immune response, cell division cycle, and cell death and survival [5, 6, 9, 10]. Messenger RNA (mRNA) and protein expression rhythms have been identified in mammalian tissues, in cell populations and in single cells [8, 11]. The molecular rhythms are redundantly controlled by both genetic circadian clocks and physiological rhythms, which build up the Circadian Timing System (CTS) [7, 12]. Mammalian CTS coordination and adjustment to environmental cycles is insured by the suprachiasmatic nuclei (SCN), a neuronal pacemaker located in the anterior hypothalamus [8].

Clock gene *Per2* is robustly and rhythmically expressed in liver and almost all mammalian tissues [13]. *In vitro* studies showed that *Per2* overexpression in malignant cells was associated with decreased cell proliferation and increased apoptosis resulting from *P53* up-regulation and the down-regulation of *Cyclin B1* (*CCNB1*), *B-cell lymphoma 2 (Bcl2)* and *c-Myc* [14–16].

Mice with constitutive *Per2* mutation (*Per*2^m/m^) were produced with a deletion in the PAS domain of *Per2* gene, and have been maintained by breeding [17]. The *Per2*^m/m^ mice, displayed an alteration of the regulatory molecular circadian clock circuitry, resulting from a rapidly degraded non-functional PER2 protein, as well as decreased apoptosis and accelerated cell cycling [18]. This might account for *Per2*^m/m^ mice developing more spontaneous and gamma-radiation-induced cancers [18, 19], and displaying enhanced susceptibility to anticancer chemotherapy [20]. Moreover, oncogene *c-Myc* was upregulated, while *P53* was down regulated in the liver of both *Per*2^m/m^ [18] and mice on chronic jet lag, with ablated *Per2* circadian transcription in liver [21] This could explain the significant increase of DEN-induced liver cancers in mice or rats exposed to constant light or iterative shifts in light-dark cycles [22, 23]. DEN carcinogenesis first involves the occurrence of dysplastic foci with altered hepatocytes. Such reversible “initiation” stage of experimental liver carcinogenesis occurs during the initial 2-4 weeks of exposure [24–26]. The hyperplastic foci then develop into neoplastic hepatocellular carcinoma nodules, following exposure to a promoter agent, or continued DEN administration for an additional 4-8 weeks [25, 26]. Liver cancer nodules subsequently progress.

Here we show that *Per2* mutation fostered three critical liver cancer hallmarks, during the initiation stage. DEN further profoundly disrupted the circadian timing system during the promotion stage. This effect was most prominent in Per2^m/m^ mice, with a further deterioration during the progression stage. Overall, *Per2* loss-of-function resulted in a fourfold increase in DEN-induced hepatocarcinoma nodules as compared to clock-proficient mice.

## RESULTS

### *Per2* control of selected liver carcinogenesis pathways

The regulatory role of clock gene Per2 was first investigated for circadian clock, proliferation, genomic instability, and inflammation, through comparing selected gene or protein circadian expressions in the liver of Wild-Type (WT) and *Per2*^m/m^ mice. Samples were obtained in non-DEN exposed animals at 6 different time points, located 4 h apart.

**Figure 1:**
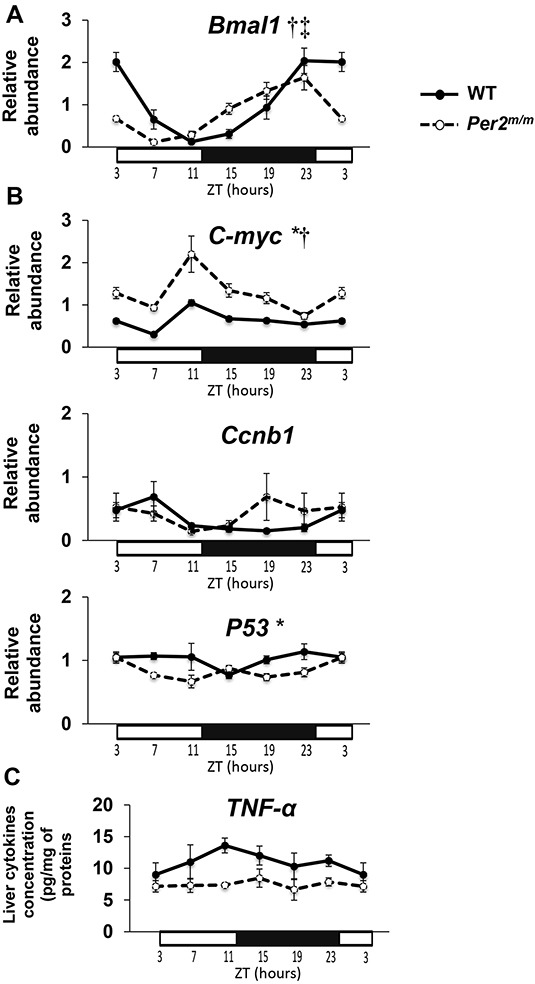
Moderation of selected liver clock, proliferation, genomic instability and pro-inflammatory cytokine patterns by *Per2* loss-of-function Each data point is mean of 5 mice at each of 6 Zeitgeber Times (ZT) for WT (dark circles) or *Per2*^m/m^ mice (open circles). Circadian changes in mean ± SEM of mRNA expression for **A**. clock gene *Bmal1;* **B**. proliferation and genomic instability genes *c-Myc, Ccnb1, and P53*; **C**. TNF-α protein concentration in liver. Results from 2-way ANOVA: * *p*(genotype)≤ 0.01; † *p*(ZT)≤ 0.01; ‡ *p*(interaction)≤ 0.01.

The mRNA expression of core clock gene *Bmal1* displayed marked and statistically significant 24 h changes both in WT and *Per2*^m/m^ mice (Figure 1A). This was also the case for all other clock genes including *Rev-erbα*, *Clock*, *Cry1*, and *Cry2* (Supplementary Figure S1A). Circadian rhythms with τ= 24 h were validated with Cosinor for each clock gene expression in each genotype, except for *Clock* in *Per2*^m/m^. Yet, circadian transcription of *Bmal1*, *Rev-erbα*, *Cry1*, and *Cry2* were phase-advanced by 2- to 5-h, and circadian amplitudes of *Bmal1, Rev-erbα*, and *Cry1* were dampened by 19% to 59% in *Per2*^m/m^ as compared to WT mice (Hotelling t-tests for phase and amplitude comparisons, *p*< 0.001 for each gene) (Supplementary Figure S1A; Table 1).

**Table 1:** Circadian rhythms of selected gene mRNA expressions and cytokine concentrations in the liver of control WT and *Per2*^m/m^ mice in the absence of any DEN exposure (Results from cosinor analysis with τ= 24h)

Function	Gene	Genotype	*p*	Mesor [95% CL]	Amplitude [95% CL]	Acrophase, ZT : min [95% CL]
Clock	*Clock*	WT	<0.001	1.03 [0.91 to 1.15]	0.56 [0.38 to 0.73]	23:35 [22:20 to 00:45]
		*Per2*^m/m^	0.13	0.97 [0.83 to 1.1]	0.19	22:25
	*Bmal1*	WT	<0.001	1.02 [0.84 to 1.19]	1.04 [0.8 to 1.28]	00:30 [23:30 to 1:30]
		*Per2*^m/m^	<0.001	0.82 [0.69 to 0.96]	0.74 [0.55 to 0.94]	20:45 [19:45 to 21:45]
	*Rev-erbα*	WT	<0.001	0.86 [0.46 to 1.25]	1.24 [0.79 to 1.72]	8:05 [6:20 to 9:55]
		*Per2*^m/m^	<0.001	1.04 [0.82 to 1.25]	1.01 [0.7 to 1.31]	5:00 [3:50 to 6:10]
	*Cry1*	WT	<0.001	1.25 [0.99 to 1.51]	1.07 [0.71 to 1.43]	21:50 [20:30 to 23:10]
		*Per2*^m/m^	<0.001	0.82 [0.7 to 0.94]	0.44 [0.27 to 0.61]	16:40 [15:10 to 18:10]
	*Cry2*	WT	0.04	1 [0.85 to 1.15]	0.25 [0.02 to 0.48]	13:35 [9:20 to 17:40]
		*Per2*^m/m^	0.04	1.05 [0.93 to 1.17]	0.22 [0.05 to 0.39]	11:40 [8:25 to 15:00]
Genome instability	*P53*	WT	0.23	1.01 [0.91 to 1.11]	0.12	3:00
		*Per2*^m/m^	0.15	0.81 [0.74 to 0.88]	0.09	1:30
	*Bcl2*	WT	0.19	0.71 [0.62 to 0.81]	0.12	5:10
		*Per2*^m/m^	0.63	0.98 [0.82 to 1.14]	0.1	22:40
	*ATM*	WT	0.02	1.16 [1.04 to 1.29]	0.25 [0.08 to 0.42]	9:00 [6:00 to 12:00]
		*Per2*^m/m^	<0.001	1.22 [1.12 to 1.32]	0.34 [0.19 to 0.49]	8:20 [6:30 to 10:10]
Proliferation	*Wee1*	WT	<0.001	1.24 [0.98 to 1.50]	1.02 [0.65 to 1.38]	13:20 [12:00 to 14:40]
		*Per2*^m/m^	<0.001	1.34 [1.15 to 1.52]	1.12 [0.86 to 1.38]	10:00 [9:00 to 11:00]
	*Ccnb1*	WT	0.004	0.32 [0.23 to 0.41]	0.24 [0.11 to 0.37]	5:40 [3:25 to 7:40]
		*Per2*^m/m^	0.25	0.41 [0.24 to 0.58]	0.19	23:20
	*c-Myc*	WT	0.046	0.64 [0.54 to 0.74]	0.17 [0.04 to 0.3]	13:40 [10:20 to 17:00]
		*Per2*^m/m^	0.038	1.27 [1.02 to 1.52]	0.46 [0.11 to 0.81]	11:40 [8:25 to 15:00]
	*K-ras*	WT	0.009	0.82 [0.72 to 0.92]	0.24 [0.09 to 0.38]	23:20 [20:50 to 25:50]
		*Per2*^m/m^	0.31	0.69 [0.6 to 0.78]	0.09	8:40
Inflammation	IL-6	WT	0.04	6.72 [5.8 to 7.64]	1.7 [0.4 to 3]	12:15 [8:50 to 15:40]
		*Per2*^m/m^	0.17	7.55 [7.14 to 7.96]	0.55	10:00
	TNF-α	WT	0.29	9.3 [8.1 to 10.5]	1.3	12:45
		*Per2*^m/m^	0.93	7.45 6.52 to 8.38	0.25	14:20

The phase advance and amplitude dampening in the liver clock of *Per2*^m/m^ translated into a 2-3 h phase advance of *c-Myc* (Figure 1B) and *Wee1* circadian rhythms (Hotelling t-test, *p*< 0.05), and the suppression of the *Ccnb1* and *K-ras* 24h rhythms as compared to WT mice (Figure 1B, Supplementary Figure S1B, Table 1). *ATM* expression displayed a similar circadian rhythm with a maximum at ZT9 and a trough at ZT19 in both genotypes. In addition, the 24-h mean mRNA expression was significantly decreased for *P53*, increased for *Bcl-2* and doubled for *c-Myc* in *Per2*^m/m^ as compared to WT mice (ANOVA, *p*≤ 0.001) (Figure 1B, Table 1).

Liver IL-6 protein concentration displayed a circadian pattern in WT with a maximum at ZT11 (Cosinor analysis, *p=* 0.04), but no such rhythm was found in *Per2*^m/m^ mice (*p*= 0.17) (Supplementary Figure S1C). The 24-h mean concentration of liver IL6 was increased in *Per2*^m/m^, although not significantly so *(p=* 0.09). No circadian change was found for liver TNF-α concentration in either genotype, yet the 24-h mean level was decreased in *Per2*^m/m^ as compared to WT mice (*p=* 0.017) (Figure 1C, Table 1).

### Relevance of clock gene *Per2* for liver carcinogenesis initiation processes

Here, the role of *Per2* was investigated on both liver pathology and selected molecular biomarkers during the initiation stage of liver carcinogenesis. WT and *Per2*^m/m^ mice received daily DEN (15 mg/kg ip) for 5 days a week over a 17 day-span (cumulative dose of 195 mg/kg). Liver samples were obtained in DEN-exposed animals at 6 different time points, located 4 h apart.

#### Pathology findings

After being on DEN for 17 days, mice displayed few hepatic lesions associated with inflammatory foci and some apoptotic and necrotic cells. The histological features were supportive of early stage carcinogenesis processes, yet no gross difference was apparent between WT and *Per2*^m/m^ mice.

#### Twenty-four hour patterns in selected gene expressions

**Figure 2:**
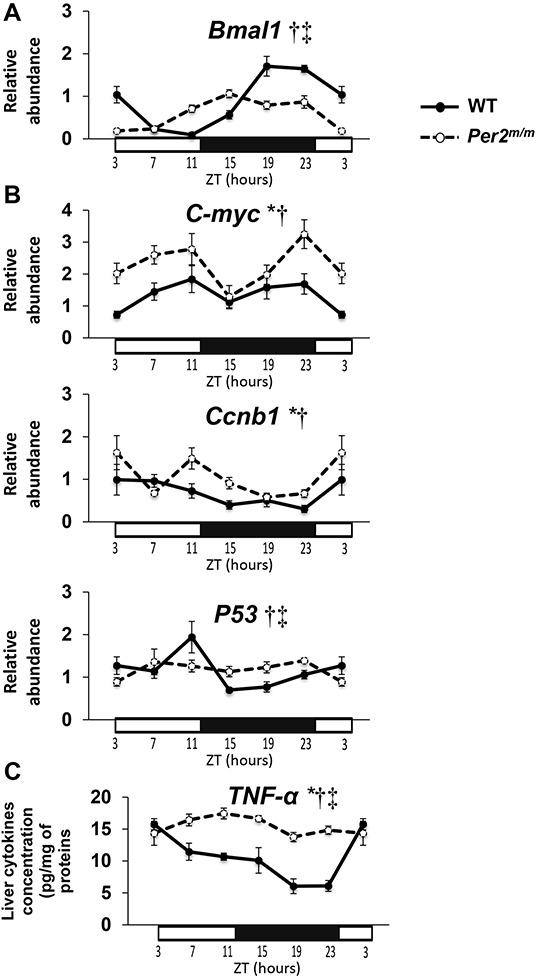
Circadian gene or protein expressions during initiation phase of carcinogenesis Each data point is mean of 5-7 mice at each of 6 Zeitgeber Times (ZT) for WT (dark circles) or *Per2*^m/m^ mice (open circles). Circadian changes in mean ± SEM of mRNA expression for **A**. clock gene *Bmal1;* **B**. proliferation and genomic instability genes *c-Myc, Ccnb1, and P53*; **C**. TNF-α protein concentration in liver. Results from 2-way ANOVA: * *p*(genotype)≤ 0.01; † *p*(ZT)≤ 0.01; ‡ *p*(interaction)≤ 0.01.

The circadian transcription patterns of the genes studied after a 17-day exposure to DEN differed markedly according to *Per2* mutation (Figure 2, Supplementary Figure S2). The mRNA expression of clock genes *Bmal1*, *Rev-erbα*, *Clock*, *Cry1*, and *Cry2* exhibited statistically significant circadian variations for both WT and *Per2*^m/m^ mice (2-way ANOVA: ZT effect, *p*< 0.001; Cosinor, *p*< 0.03 to *p*< 0.001). The rhythms were phase advanced by 5 h for *Bmal1* (Figure 2A), by 4 h for *Clock, Cry1* and *Cry2*, and by 7 h for *Rev-erbα* in *Per2*^m/m^ as compared to WT (Supplementary Figure S2A, p< 0.001 for each gene). The circadian amplitudes of clock genes were also reduced by 34 to 74% except for *Cry2* in *Per2*^m/m^ compared to WT (*p* <0.001) (Supplementary Figure S2A, Table 2).

**Table 2:** Circadian rhythms of selected gene mRNA expressions and cytokine concentrations in the liver of WT and *Per2*^m/m^ mice exposed to DEN over 17 days (Results from cosinor analysis with τ= 24h)

Function	Gene	Genotype	*p*	Mesor [95% CL]	Amplitude [95% CL]	Acrophase, ZT : min [95% CL]
Clock	*Clock*	WT	<0.001	0.96 [0.89 to 1.03]	0.41 [0.29 to 0.53]	21:50 [20:40 to 23:00]
		*Per2^m/m^*	<0.001	0.85 [0.77 to 0.93]	0.27 [0.15 to 0.39]	17:40 [15:55 to 19:25]
	*Bmal1*	WT	<0.001	0.87 [0.76 to 0.98]	0.89 [0.73 to 1.05]	21:45 [21:00 to 22:30]
		*Per2^m/m^*	<0.001	0.64 [0.55 to 0.73]	0.42 [0.3 to 0.54]	17:00 [15:50 to 18:10]
	*Rev-erbα*	WT	<0.001	0.74 [0.57 to 0.91]	0.85 [0.61 to 1.09]	7:20 [6:20 to 8:20]
		*Per2^m/m^*	<0.001	0.71 [0.63 to 0.79]	0.39 [0.28 to 0.5]	00:10 [23:10 to 1:10]
	*Cry1*	WT	<0.001	1 [0.88 to 1.12]	1.03 [0.86 to 1.2]	20:30 [19:50 to 21:10]
		*Per2^m/m^*	<0.001	0.49 [0.43 to 0.55]	0.27 [0.19 to 0.35]	16:40 [15:30 to 17:50]
	*Cry2*	WT	0.03	0.93 [0.84 to 1.02]	0.17 [0.05 to 0.29]	9:10 [6:00 to 12:20]
		*Per2^m/m^*	<0.001	0.84 [0.78 to 0.90]	0.18 [0.1 to 0.26]	5:35 [3:45 to 7:35]
Genome instability	*P53*	WT	0.04	1.14 [0.94 to 1.34]	0.38 [0.1 to 0.66]	7:40 [4:20 to 11:00]
		*Per2^m/m^*	0.91	1.02 [1.06 to 1.34]	0.04	14:45
	*Bcl2*	WT	0.08	0.93 [0.78 to 1.08]	0.26	22:50
		*Per2^m/m^*	0.06	1.11 [1 to 1.22]	0.2	23:30
	*ATM*	WT	0.007	1.12 [0.99 to 1.25]	0.32 [0.14 to 0.5]	18:50 [16:30 to 21:10]
		*Per2^m/m^*	0.32	1.06 [0.94 to 1.18]	0.12	18:50
Proliferation	*Wee1*	WT	0.009	0.97 [0.78to 1.17]	0.42 [0.14 to 0.70]	13:10 [10:40 to 15:40]
		*Per2^m/m^*	0.09	1.15 [0.95 to 1.35]	0.31	5:10
	*Ccnb1*	WT	0.02	0.64 [0.48 to 0.8]	0.33 [0.11 to 0.55]	6:40 [3:40 to 9:40]
		*Per2^m/m^*	0.19	0.99 [0.77 to 1.21]	0.28	7:00
	*c-Myc*	WT	0.79	1.4 [1.12 to 1.68]	0.13	15:30
		*Per2^m/m^*	0.43	2.33 [1.95 to 2.71]	0.34	3:20
	*K-ras*	WT	<0.001	0.88 [0.78 to 0.97]	0.39 [0.25 to 0.52]	21:20 [19:50 to 22:40]
		*Per2^m/m^*	0.006	0.84 [0.73 to 0.94]	0.26 [0.11 to 0.41]	19:00 [16:30 to 21:30]
Inflammation	IL-6	WT	0.001	7.9 [7.1 to 8.7]	2.36 [1.18 to 3.54]	10:25 [8:30 to 12:20]
		*Per2^m/m^*	<0.001	9.5 [8.9 to 10.1]	1.7 [0.9 to 2.5]	12:00 [10:00 to 14:00]
	TNF-α	WT	0.004	10 [8.6 to 11.4]	3.5 [1.5 to 5.5]	6:40 [4:30 to 8:50]
		*Per2^m/m^*	0.023	15.57 [14.73 to 16.41]	1.7 [0.5 to 2.9]	10:45 [7:50 to 13:40]

Twenty four-hour changes were also statistically validated for the proliferation genes *c-Myc*, as wells as *Wee1*, *Ccnb1*, and *K-ras*, and for the genomic instability genes *P53, ATM*, and *Bcl2* (Figure 2B, Supplementary Figure S2B) (2-way ANOVA: ZT effect, *p*< 0.01 for each gene). Circadian rhythms were validated with Cosinor for *Wee1, Ccnb1, K-ras*, *ATM*, and *P53* in WT mice (*p*< 0.05 to *p*< 0.001), but only for *K-ras* in *Per2*^m/m^ (*p*= 0.006), while 12-h rhythms were found for *c-Myc* in both for WT and *Per2*^m/m^ (*p*= 0.02 and *p*= 0.005, respectively), as well as for *Wee1, Ccnb1, ATM, Bcl-2* in *Per2*^m/m^ (*p*≤ 0.03 for each gene). *Per2* mutation was also associated with increased daily average mRNA expression of *c-Myc* by 66%, *Ccnb1* by 55%, and *Bcl-2* by 20% (*p*< 0.04 to *p*< 0.001) (Figure 2B, Table 2).

Liver IL-6 and TNF-α concentrations displayed marked 24-h changes in both genotypes (2-way ANOVA: ZT effect, *p*< 0.001; Cosinor with τ = 24*h*, *p*= 0.02 to < 0.001) (Figure 2C, Supplementary Figure S2C, Table 2). Both mean IL-6 and TNF-α concentrations were significantly increased in *Per2*^m/m^ as compared to *WT* (*p*= 0.003 and *p*< 0.001, respectively).

#### DEN-induced changes during carcinogenesis initiation according to Per2 mutation

Overall, the 17-day exposure to DEN markedly altered the circadian gene expression patterns, and did more so in the *Per2*^m/m^. In WT mice, DEN phase-advanced the mRNA expression patterns of the five clock genes and that of *K-ras* by 1 to 4 h, and nearly inverted the *ATM* rhythm, as compared to untreated controls (Hotelling t-test for phases: *p*= 0.01 to *p*< 0.001). DEN dampened the circadian amplitude of *Bmal1*, *Rev-erbα, Clock, Cry2* and *Wee1* by 15 to 59% and amplified the 24-h rhythms of *Ccnb1, K-Ras* and *ATM* by 28 to 63% (Hotelling t-test for amplitude: *p*= 0.04 to *p*< 0.001) (Figures 1 and 2, and left panels in Supplementary Figures S1 and S2).

In *Per2*^m/m^, DEN treatment for 17 days phase-advanced the 24-h rhythms in *Bmal1, Rev-erbα* and *Cry2* by 4 to 6 hours (Hoteling t-test for phases, *p*< 0.001), shortened the rhythm period from 24 h to 12 h for *c-Myc, Wee1* and *Ccnb1*, and induced a 24-h rhythm for *K-ras* (*p*= 0.03 to *p*= 0.005) as compared to untreated Per2^m/m^ controls. The 24 h amplitude was reduced by 43% for *Bmal1*, 61% for *Rev-erbα* and 39% for *Cry1* as compared to untreated controls (Hotelling t-test for amplitudes: *p*< 0.001 for each gene) (Figures 1 and 2, and right panels in Supplementary Figures S1 and S2).

DEN exposure significantly reduced the 24-h mean expression of *Bmal1, Rev-erbα, Cry1* and *Cry2* by 20 to 40% in *Per2*^m/m^ and only that of *Cry1* by 20% in WT. In contrast, DEN exposure nearly doubled the 24-h mean expression of *c-Myc* and that of *Ccnb1* both in WT and *Per2*^m/m^ with regard to their respective controls (*p*< 0.05 to *p*< 0.001) (Table 2).

#### Severe circadian disruption during carcinogenesis initiation stage in *Per2*^m/m^ on DEN

The rest-activity and temperature circadian patterns were monitored using implanted telemetry sensors in WT and Per2^m/m^ mice for 17 days before DEN exposure, and throughout the 17-day DEN-initiation stage of liver carcinogenesis. The circadian rhythm in plasma corticosterone was determined at completion of this initiation stage in DEN-treated mice and in untreated controls.

**Figure 3:**
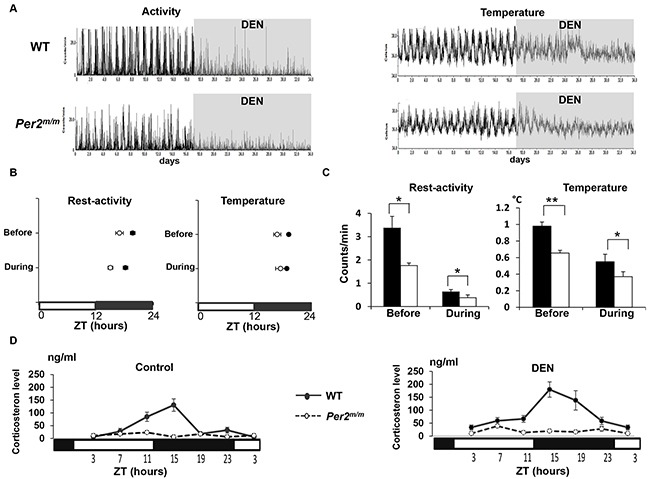
DEN effects during initiation phase on circadian rhythms in rest-activity, body temperature and corticosterone rhythms according to Per2 mutation during early carcinogenesis **A**. Chronograms of telemetered rest-activity (left) and body temperature (right) in WT and *Per2*^m/m^ before and during daily DEN exposure for 17 days. **B**. Corresponding mean circadian acrophases in WT (dark circles) and *Per2*^m/m^ (open circles). **C**. Corresponding mean circadian amplitudes in WT (dark boxes) and *Per2*^m/m^ (open boxes). **D**. Twenty-four hour changes in mean plasma corticosterone concentration (±SEM) in untreated WT and *Per2*^m/m^ mice (left panel) and in WT and *Per2*^m/m^ mice after a 17-day DEN exposure (right panel). *p <0.05, **p <0.01.

Both rest-activity and body temperature patterns were markedly dampened during DEN exposure with more severe apparent alteration in *Per2*^m/m^ as compared to WT over the 17-days of DEN exposure (Figure 3A).

Cosinor analysis documented an acrophase advance by nearly 2 h and 30 min in *Per2*^m/m^ for both rest-activity (ZT17:00 *vs* ZT19:20, *p*= 0.014) and body temperature (ZT16:50 *vs* ZT19:30, *p*= 0.015) as compared to WT mice, before DEN administration (Figure 3B). The circadian amplitude was nearly halved for rest-activity (*p*= 0.03) and reduced by nearly 40% for body temperature (*p*= 0.009) in *Per2*^m/m^ as compared to WT mice (Figure 3C). DEN exposure did not modify the circadian temperature acrophases, yet it induced 1-h phase advance for rest-activity in WT and a 2-h phase advance in *Per2*^m/m^ (Hotelling t-test for phase comparisons, *p*= 0.004) (Figure 3B).

DEN reduced the circadian amplitude of rest-activity by nearly 5.3-fold and 4.6-fold in WT and *Per2*^m/m^ respectively (Hotelling t-test, *p*= 0.03) and halved that of temperature in both groups (Hotelling t-test for amplitudes, *p*= 0.02) (Figure 3C).

Plasma corticosterone was rhythmic in WT mice (Cosinor, *p*< 0.001), but not in *Per2*^m/m^ both in untreated controls and in mice exposed to DEN, with 4.3-fold lower 24-h mean corticosterone levels in treated *Per2*^m/m^ as compared to WT (*p*< 0.001) (Figure 3D).

### Liver carcinogenesis according to *Per2* mutation

**Figure 4:**
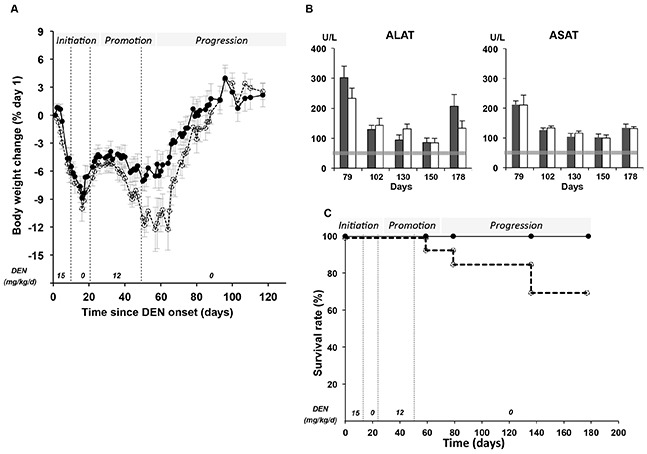
Systemic toxicity of DEN according to *Per2* loss-of-function in the initiation, promotion and progression stages **A** Relative body weight changes (mean ± SEM) in WT (dark circles) and *Per2*^m/m^ mice (open circles) during and after DEN exposure. The daily DEN doses (mg/kg) are shown in italic, for corresponding exposure spans. **B** Histogram of serum levels of ALAT and ASAT (mean ± SEM) after DEN exposure in WT (dark box) and *Per2*^m/m^ (open box) mice in the progression stage. Mean ± SEM ALAT and ASAT in untreated control mice is represented by the gray horizontal bar. **C** Survival rate in WT (dark circles) and *Per2*^m/m^ (open circles) mice exposed to DEN over 180 days.

Subsequently, the role of *Per2* was investigated regarding DEN-induced liver carcinogenesis from initiation to promotion and progression. WT and *Per2*^m/m^ mice received daily DEN (15 mg/kg ip) for 5 days per week over 12 days followed with a 9-day DEN-free span. This allowed for the recovery from sustained body weight loss, during this initiation carcinogenesis stage (cumulative dose = 150 mg/kg) (Figure 4A). A reduced dose of DEN (12 mg/kg/day) was then administered according to the same 5-days on/2-days off schedule over 29 days during this promotion stage (cumulative dose = 252 mg/kg). Overall, the mice received a cumulative dose of 402 mg/kg of DEN over a 50 day-span. They were euthanized after allowing for a 129-day span, corresponding to the carcinogenesis progression stage for histopathological analyses.

#### Increased DEN toxicity during carcinogenesis promotion stage in *Per2*^m/m^ mice

The DEN daily dose had to be reduced from 15 to 12 mg/kg, because of a rapid body weight loss during the carcinogenesis initiation stage, irrespective of *Per2* mutation (8.9 ± 1.3 % in WT *vs* 10 ± 1.4 % in *Per2*^m/m^, *p*= 0.55). Following DEN re-exposure during the promotion stage, mean body weight loss reached a second nadir 57 days after DEN treatment onset, which was ∼twice as large in *Per2*^m/m^ mice as compared to WT (12.3 ± 2.2 % *vs* 6.5 ± 1.3 %, *p* from ANOVA < 0.001). Complete recovery of body weight was also slower in the *Per2*^m/m^ as compared to WT (38 days *vs* 26 days) (Figure 4A). The aspartate levels of both alanine aminotransferase (ALAT) and aspartate aminotransferase (ASAT) were increased by up to six- and four-fold respectively ∼30 days after completion of DEN exposure as compared to untreated controls. However, no difference was found according to *Per2* mutation over the 90-day span corresponding to the carcinogenesis progression stage (Figure 4B). No toxic death was encountered in the WT mice on DEN. In contrast, four *Per2*^m/m^ mice died with severe liver alterations before study completion (*p* from log Rank= 0.009) (Figure 4C). Two mice displayed histological evidence of liver inflammation, oval cell types, with major dysplasia and enlarged nuclei, associated with severe body weight loss (37.4%) or ascites on days 59 and 79 respectively. The liver histology of both other mice revealed inflammatory foci. In addition, precancerous dysplasia was found for that mouse dead on day 136, while 4 liver cancer nodules were identified in the mouse that died on day 137. Only, this latter animal was counted among those that developed liver cancer.

#### Major DEN-induced circadian disruption in *Per2*^m/m^ mice

**Figure 5:**
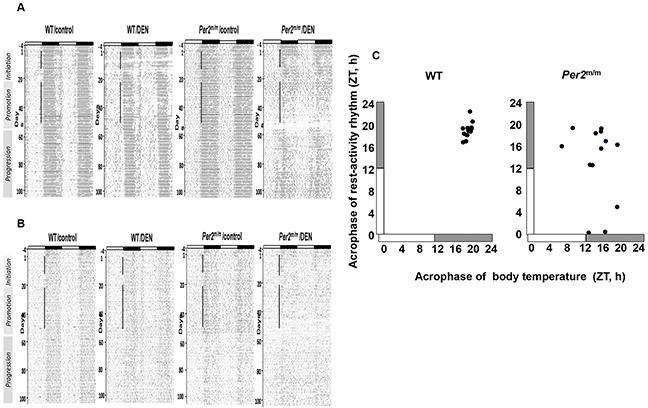
Examples of circadian physiology records in mice according to *Per2* loss-of-function and DEN exposure for 50 days (initiation and promotion stages) Representative double-plots of temperature **A**. and activity **B**. rhythms according to *Per2* loss-of-function and DEN exposure. The vertical black lines indicate exposure to DEN or vehicle (control) for 7 weeks. **C**. Relation between acrophases of rest-activity and body temperature rhythms 7 weeks after DEN exposure onset in individual WT mice (left) and *Per2*^m/m^ mice (right). Note robust coordination in WT and internal desynchronization in *Per2*^m/m^.

The rest-activity and temperature circadian patterns were monitored in WT and *Per2*^m/m^ mice for 5 days before DEN exposure, and throughout the initiation, promotion and progression stages of liver carcinogenesis using implanted telemetry sensors (Exp III), in order to assess the dynamics of circadian physiology disruption. Double-plot representations of both circadian biomarkers in individual mice revealed that the circadian disruption produced by DEN was transient for WT, and sustained for *Per2*^m/m^ mice. The temperature rhythm appeared to be more sensitive to disruption as compared to rest-activity, and this was especially true during the progression carcinogenesis stage (Figure 5A and 5B). Moreover, a significant correlation was found between the circadian acrophases of both rhythms for WT mice (r= 0.571, *p*= 0.042) but not for *Per2*^m/m^ individuals (r= 0.197, *p*= 0.518). This finding supports the coupling of both physiological rhythms in WT and the occurrence of internal desynchronization in *Per2*^m/m^ as a result of DEN exposure (Figure 5C).

**Figure 6:**
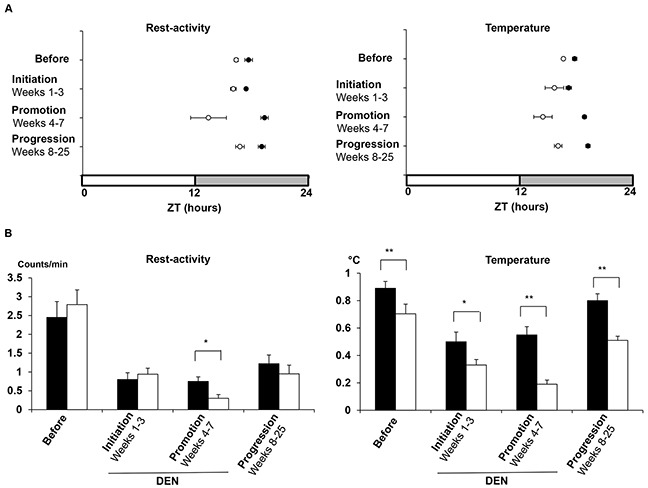
Dynamics of rest-activity and body temperature rhythms throughout liver carcinogenesis processes according to *Per2* loss-of-function **A**. Acrophase charts for rest-activity (left panel) and body temperature (right panel). Each acrophase (time of maximum in best-fitting 24-h cosine function) is displayed with its 95% C.L. for WT (dark circles) and *Per2*m/m (open circles) before, during the initial 3 weeks (weeks 1-3; initiation stage) and the subsequent 4 weeks on DEN (weeks 4-7; promotion stage), and after exposure (weeks 8-25; progression stage). **B**. Corresponding histograms of 24h mean amplitudes with 95% C.L. for rest- activity (left panel) and body temperature (right panel) in WT (dark boxes) and *Per2*m/m (open boxes). * *p*< 0.05; ** *p*< 0.001.

Cosinor analysis further indicated that both rest-activity and temperature patterns displayed periodic 24-h changes in *Per2*^m/m^ and WT mice for the 5 days preceding DEN exposure. A 1-h phase advance was statistically validated in *Per2*^m/m^ for both rest-activity (ZT16:07 *vs* ZT17:07, Hotelling t-test, *p*= 0.016) and body temperature (ZT16:39 *vs* ZT17:45, *p*= 0.001) (Figure 6A). Although the baseline rest-activity amplitude was similar in both genotypes, that of body temperature was significantly lower in *Per2*^m/m^ (0.7 *vs* 0.88°C, Hotelling T test, *p*< 0.001) (Figure 6B).

DEN suppressed the circadian rhythm in rest-activity for 2/13 WT (15%) and 8/13 *Per2*^m/m^ (62%), and that in body temperature for 2/13 *Per2*^m/m^ (15%) during the initiation and/or the promotion stages. In the WT mice with circadian rhythms, DEN reduced ∼three-fold the circadian amplitude of rest-activity and halved that of body temperature as compared to baseline. Such effect was achieved during the carcinogenesis initiation stage for both genotypes. During the promotion stage however, the amplitudes in rest-activity and body temperature deteriorated down to 1/9 and 1/4 of their respective baseline values in *Per2*^m/m^ mice while no further changes were noticed in WT (*p*< 0.001) (Figure 6B). Over this 4-week span, the circadian phase was delayed by ∼2 h for rest-activity and by 1 h for body temperature as compared to baseline in WT (Paired T-tests, *p*= 0.01 and *p*= 0.003, respectively). In contrast both of these rhythms were phase-advanced by 3 h and 2 h respectively in *Per2*^m/m^ (Paired t-test, *p*= 0.04) (Figure 6A).

#### Accelerated progression of liver carcinogenesis in *Per2*^m/m^ mice

**Figure 7:**
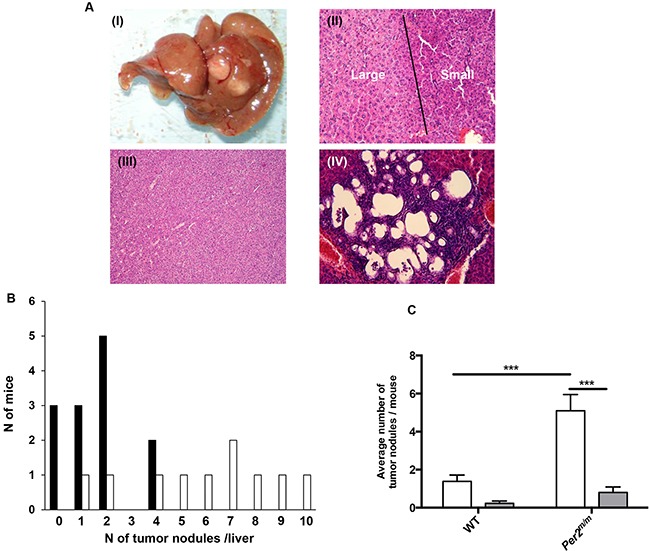
DEN-induced liver alterations according to *Per2* loss-of-function found on day 178 **A**. Typical macroscopic view of a deeply reorganized liver with cancer nodules arising in a *Per2^m/m^* mouse (**I**); Typical microscopic view of large (left) or small (right) cell dysplasia coexisting in the same liver sample of a *Per2*^m/m^ mouse and separated by a dark marker line (**II**); Microscopic view of hepatocarcinoma (**III**), and cholangiocarcinoma (**IV**). **B**. Number of tumor nodules per mouse liver in WT (black boxes) and *Per2*^m/m^ (open boxes). **C**. Average number of hepatocarcinomas (open box) or cholangiocarcinomas (dark box) in WT and *Per2*^m/m^ mice. *** *p*< 0.001.

All the mice were euthanized and inspected for liver, lung, kidney or other tumor deposits 178 days after the onset of the DEN administration protocol, i.e. at an advanced carcinogenesis progression stage. Macroscopic tumor nodules were observed in all *Per2*^m/m^ mice [Figure 7A (I)]. Histological analysis revealed the occurrence of minor or severe liver dysplasia [Figure 7A (II)], as well as both hepatocellular carcinoma (HCC) and cholangiocarcinoma (CCA) [Figure 7A (III-IV)], and pre-neoplastic nodules, inflammatory infiltrations and apoptotic hepatocytes. DEN further induced primary lung tubulo-papillary tumor in 5 WT and 3 *Per2*^m/m^ with associated inflammatory foci. A single HCC metastasis was found in the kidney of a WT mouse.

Histopathological analysis was performed on three parallel transverse liver sections equispaced by 1 mm. Liver tumor nodules, with diameters ranging from 1 to 11 mm, were found in all the *Per2*^m/m^ and in 77% of the WT mice. Histology revealed ∼six times as many HCC as compared to CCA (69 *vs* 11), in both groups. The average number of tumors per animal, including HCC and CCA, was 3.7-fold as high in *Per2*^m/m^ as compared to WT (Figure 7B) (Kruskal-Wallis ANOVA, *p*= 0.01). The number of tumor nodules ranged from 0 to 4 in WT mice, and from 1 to 10 in *Per2*^m/m^. As a result, the proportion of mice with more than 2 tumor nodules was ∼five-fold as high in *Per2*^m/m^ as compared to WT (80% *vs* 15 %, Fisher exact test, *p*= 0.003). The average number of HCC per mouse was 3.7-fold as high in *Per2*^m/m^ mice as compared to WT (Kruskal-Wallis ANOVA, *p*< 0.001) (Figure 7C).

## DISCUSSION

To our knowledge, this study is the first one that reveals the crucial role of clock gene *Per2* in liver carcinogenesis. DEN was used here as a carcinogen for both initiation and promotion of primary liver cancers in mice. Mutation of clock gene *Per2* increased nearly fivefold the rate of mice with two or more primary liver cancers as compared to WT exposed to the same DEN dose. Previous studies demonstrated that mutation or knock out of clock genes *Per2, Cry1/Cry2* or *Bmal1* also accelerated the development of lymphomas following whole body exposure to γ radiations [18, 19]. The clinical relevance of our findings is illustrated by the fact that *Per2* expression was also decreased in human HCC as well as in other human cancers, as compared to the corresponding healthy tissues [27, 28]. Furthermore, the down regulation of *Per2* expression in human cancers was usually associated with poor patient outcomes [29]. In contrast, the overexpression of *Per2* inhibited proliferation and arrested cell cycling in cancer cell lines, thus supporting the negative growth-regulatory properties of *Per2* [16].

Circadian disruption resulting from an altered light-dark environment had been previously identified as a promoter of liver carcinogenesis in rats and mice [22, 23]. In contrast, the current study showed that initiation, promotion and progression stages of liver carcinogenesis were regulated by *Per2*. Indeed, Per2 mutation resulted in profound circadian disruption of selected gene and protein expressions during the initiation stage, although no apparent difference was observed at histopathologic analysis of liver obtained after 17 days on DEN. Prior to DEN exposure, *Per2* mutation was already associated with profound molecular circadian disruption in liver despite regular light-dark exposure. Thus, both 24-h expressions and circadian amplitudes of *Bmal1* and *Cry1* were markedly reduced. In addition, a 2- to 5-h phase advance was shown for clock and cell cycle-related genes, supporting an alteration of the clock-cell cycle coupling [10] in Per2^m/m^. The 24-h mean mRNA expression of *c-Myc* was doubled in the liver of untreated *Per2*^m/m^ as compared to WT mice in the current study. This was related to the demonstrated role of *Bmal1* as a repressor of proto-oncogene *c-Myc* through binding to its P1 promoter [18].

The several-fold higher rate of liver cancers in *Per2*^m/m^ mice as compared to WT at ∼6 months after the beginning of treatment was preceded by the up-regulation of several critical liver carcinogenesis pathways at 17 days after DEN administration onset. Thus, molecular markers of proliferation, genomic instability, apoptosis, and inflammation were significantly deregulated in *Per2*^m/m^, as compared to WT, despite apparently similar extent of histological liver inflammation. DEN exposure further significantly dampened the circadian transcription amplitude of *Bmal1, Rev-erbα, Cry1*, and *Clock* in *Per2*^m/m^ as compared to WT. This severe alteration of the liver clock translated into the up-regulation and/or the circadian deregulation of the selected molecular markers of cellular proliferation including *c-Myc, Ccnb1, Wee1, and K-ras*. DEN exposure had less prominent effects on the 24-h expression patterns of the selected genomic instability genes *ATM* and *P53* both in WT and *Per2*^m/m^. In contrast, apoptosis induction was reported in cultured mouse primary hepatocytes exposed to DEN for 24 h, an effect which was suppressed in *Clock* mutant hepatocytes [30].

DEN exposure in the initiation stage has long been known as inducing hepatocyte deaths, activation of NF-κB pathway in liver macrophages, and liver inflammation [31, 32]. In our study, histologic lesions were documented at 17 days, with occasional deaths, occurring in *Per2*^m/m^ mice over the subsequent 3 months. IL-6 and TNF-α proteins were significantly increased in *Per2*^m/m^ as compared to WT, while their 24-h amplitudes were less in *Per2*^m/m^. During the initiation of carcinogenesis, NF-κB overexpression accelerates cellular proliferation and inhibits apoptosis through the activation of *IL-6* and *TNF-α* genes [33, 34]. Indeed, *Per2*^-/-^ mice, a construct which differed from *Per2*^m/m^ by the lack of any PER2 protein, also displayed increased *TNF-α* expression jointly with liver cholestasis or fibrosis, in response to physical liver injury [35].

DEN metabolism also involves clock-controlled pathways, which were not explored here. DEN was administered at ZT11, near the end of the light span, coincidently with the physiologic peak in plasma corticosterone in WT mice. This hormone triggers the bioactivation of DEN by hepatic microsomes, thus potentiates its liver toxicity [36]. DEN is mainly bioactivated into ethyldiazonium ion by CYP2E1 and CYP2A5 in mouse liver [24]. *Cyp2e1* displayed mRNA and protein circadian variations in mouse liver, with peak expression occurring in the late light span (ZT10), for mRNA, and in the early dark span (ZT15), for protein. Moreover, *Cyp2e1* activation was suppressed by PER2 [37]. Thus, *Per2* mutation would expectedly enhance DEN bioactivation. However, clock-controlled glutathione-related genes in liver also peaked in the late light span, resulting in highest levels of reduced glutathione near ZT15 [38]. This supported highest DEN detoxification near the middle of the activity phase of mouse rest-activity circadian rhythm. Taken together, the available data revealed that DEN bioactivation and detoxification pathways were both controlled by the circadian clock in mouse liver. However, these opposite processes followed a coincident circadian pattern, thus suggesting their respective effects on DEN could antagonize each other. The plasma levels of liver ASAT and ALAT were increased up to six-fold as compared to controls at the beginning of the progression carcinogenesis stage, then decreased over about 2 months, then increased again. These changes reflected the complex dynamics in the underlying DEN-induced chronic cirrhosis and fibrosis, which constitute the grounds on which liver cancers develop. Since no difference was found according to Per2 mutation, this supports the hypothesis that *Per2* played a critical role for the development of liver cancers rather than for that of chronic liver disease.

The deleterious effects of DEN on circadian clocks during carcinogenesis initiation was also documented here using circadian biomarkers. Both rest-activity and core body temperature rhythms displayed severe disruption in WT mice, while an internal desynchronization was found in *Per2^m/m^*. We hypothesized that the release of inflammatory cytokines resulting from initial acute liver damage could in turn disrupt circadian physiology, thus depriving molecular clocks and clock-controlled pathways in peripheral tissues from resetting time cues. Such desynchronization of molecular clocks could further alter xenobiotic metabolism and detoxification, and favor carcinogenic processes, and even more so in clock-defective *Per2^m/m^* mice [39, 40]. Circadian disruption remained consistently worse in *Per2*^m/m^ as compared to WT mice during both the promotion and the progression stages of liver carcinogenesis. Such profound and sustained alterations in the circadian timing system thus contributed to enhance cancer development throughout all three carcinogenesis stages.

In summary, the loss of function of *Per2* critically deregulated the circadian clock and clock-controlled pathways, whose alteration contributed to three cancer hallmarks, within the initial 3 weeks of DEN exposure. As a result, the formation of liver cancers was increased by up to four-fold, six months after the onset of carcinogen exposure, Clock gene *Per2* could thus represent a promising target for liver cancer prevention and therapy.

## MATERIALS AND METHODS

### Mice

Animal experiments were performed under the guidelines approved for animal experimental procedures by the French Ethical Committee (decree 87-848). Male 129SvEv^Brd^ / C57BL/6-Tyrc-Brd mice with or without constitutive *Per2* mutation were used for all experiments [17]. The *Per2*^m/m^ mice and their WT counterpart were a generous gift from Urs Albrecht (Freiburg, Switzerland). They were kept at 21°C to 23°C in chronobiologic facilities, with light intensity at cage level ranging from 240 to 580 lux according to cage location. Mice were aged 8 to 14 weeks upon inclusion in each experiment, with 85% of them being 10-14 weeks old (Supplementary Table S1). They were stratified according to age, then allocated to treatment group, and synchronized with an alternation of 12 h of light (L) and 12 h of darkness (D) (LD 12:12) for at least 3 weeks before starting and during each experiment, and had free access to food and water. Zeitgeber Time (ZT) 0 corresponded to light onset, while ZT12 corresponded to dark onset.

### Experimental design

The study involved three experiments (Exp). Exp I aimed at jointly determining the regulatory role of *Per2* gene on circadian clock, proliferation, genomic instability, and inflammation both in the absence of DEN administration and during the carcinogenesis initiation stage, after 17-day DEN treatment. Thirty WT and 30 *Per2*^m/m^ mice were euthanized at 6 time points, located 4 h apart, i.e. ZT3, 7, 11, 15, 19, and 23. Blood (200 μL) was sampled on heparin in order to determine plasma corticosterone concentration. A liver lobe was obtained and frozen in liquid nitrogen, then kept at -80°C. Total RNA and proteins were extracted from each mouse liver for the determination of selected gene mRNA expressions, as well as selected cytokine concentrations.

Exp II assessed the role of *Per2* on liver pathology (IIA) and several main molecular mechanisms (IIB) during the initiation stage of liver carcinogenesis. For Exp IIA, 6 WT and 6 *Per2*^m/m^ mice received daily intraperitoneal injection (i.p) of DEN (Sigma-Aldrich, Saint Quentin Fallavier, France) at ZT11 for 17 days (cumulative dose of 195 mg/kg). Twenty-four hours after the last injection, mice were sacrificed and livers were removed for histological studies.

For Exp IIB, 30 WT and 42 *Per2*^m/m^ mice, received a cumulative dose of 195 mg/kg DEN (15 mg/kg/d i.p. at ZT11) over a 17-day span. Five WT and 5 *Per2*^m/m^ mice underwent circadian monitoring of rest-activity and body temperature for 5 days before and during DEN exposure. Plasma corticosterone concentration and selected liver gene mRNA expressions, as well as liver IL-6 and TNF-α concentrations were determined in 5 WT and 7 *Per2*^m/m^ every 4h for 24h.

Exp III determined the role of *Per2* mutation in DEN-induced liver carcinogenesis, involving all three stages. Thirteen WT and 13 *Per2*^m/m^ mice received a cumulative dose of 402 mg/kg of DEN, using a 5-days on and 2-days off schedule over an overall exposure duration of 50 days. Two control mice per genotype received 0.9% NaCl, solution. DEN was injected i.p. to mice aged 12-14 weeks at ZT11 from day 1 to 12 at a daily dose of 15 mg/kg, followed with a 9-day DEN-free interval, and DEN (12 mg/kg daily) from day 22 to 50. Body weight was recorded daily at ZT11 before each injection for 50 days, then every 3 days for 128 days. Mortality was checked daily. Blood (100 μl) was sampled from the retro-orbital sinus on days 79, 102, 130, 150, and 178, i.e. during the carcinogenesis progression stage, for the determinations of ASAT and ALAT concentrations, with a Synchron LX20 Clinical System (Beckman Coulter, Villepinte, France). All the mice underwent monitoring of rest-activity and core body temperature for 5 days before DEN exposure, throughout DEN treatment, and subsequently until experiment completion. Mice were euthanized 178 days after DEN administration onset for pathology and histology studies.

### Circadian rest-activity and core body temperature rhythm monitoring

Mice underwent i.p. implantation of a telemetry sensor and radiotransmitter under isoflurane anesthesia 2 weeks before starting effective recording of locomotor activity and body temperature every 10 min (Physio Tel, TA 10 TA-F20, Data Sciences, St. Paul, Minnesota) (39). For Exp II, mice were monitored for 17 days before DEN treatment onset, and during the 17-day carcinogenesis initiation stage. For Exp III, body temperature and rest-activity were recorded for 5 days before DEN administration and until completion of experiment, i.e. 178 days after DEN exposure onset.

### Pathology and histological analyses

Gross pathology examination was performed on the 12 WT and *Per2*^m/m^ mice for Exp IIA and on all the 26 DEN-exposed WT and *Per2*^m/m^ mice included in Exp III, in order to identify any macroscopic abnormality either after intercurrent death or following sacrifice 17 days for Exp IIA and 178 days for Exp III after DEN exposure onset. Organs sampled involved whole liver in Exp IIA and whole liver, both lungs, and both kidneys in Exp III. They were removed immediately, then fixed in 4% formaldehyde for 24 h, then dehydrated and embedded into paraffin. Three 4 μm-thick serial histological sections were obtained 1 mm apart in each liver, and stained with hematoxylin-eosin-saffron. A senior pathologist (C.G.) carefully examined each slide under a light microscopic for counting all detectable tumor nodules, for measuring their diameter, and for identifying their histological characteristics, without any information on genotype or experimental conditions.

### RNA extraction, cDNA synthesis, and RT-qPCR

Total liver RNA was isolated from the frozen tissue specimens with an acidic solution containing acid guanidinium thiocyanate, phenol, and chloroform (41). cDNA were synthesized using SuperScript Reverse Transcriptase kit (Invitrogen, Cergy Pontoise -France). Quantitative PCR (qPCR) was performed in a light cycler system instrument using Light Cycler 480 SYBR Green I Master kit (Roche, France). All primers were obtained from Invitrogen Life Technologies (Supplementary Table S2).

### Plasma corticosterone and liver cytokines determinations

Blood (200 μL) was sampled on heparin from the retro-orbital sinus prior to mouse euthanizing. Blood was centrifuged at 5000 rpm, plasma extracted and frozen at -80°C. Plasma corticosterone concentration was determined with ELISA (Enzo Life Sciences, Villeurbanne-France). Data were expressed as ng/mL of plasma.

Liver proteins were extracted with an established buffer (Tris 20 mM, EDTA 2 mM, EGTA 5 mM, DTT 0.5 mM, NaCl 100 Mm, and Triton 0.1%). The liver concentrations were determined with “BioPhotometer” for total proteins, and with commercially available ELISA kits (Invitrogen, Cergy Pontoise-France) for IL-6 and TNF-α. Cytokine concentrations in liver were expressed as pg/mg of proteins.

### Statistical analyses

Mean ± standard error of the mean (SEM) were computed for all quantitative data according to sampling time (ZT), genotype (WT *vs Per2*^m/m^), experimental condition (DEN exposure vs no DEN). Group comparisons involved multiple way analyses of variance (ANOVA) with Scheffé's contrast tests. Incidence data were compared with Chi-Square or Exact Fischer test.

Survival curves were compared with log-rank. Time series were first examined with chronogram inspection. Rest-activity and temperature time series were analyzed with spectral analysis through Fast Fourier Transform in order to determine the occurrence of a dominant period τ in the circadian domain. Cosinor analyses with τ= 24h were performed for all the 24h time series in order to determine mesor (24h mean), amplitude (half the difference between maximum and minimum value of fitted cosine function) and acrophase (timing of maximum, referred to ZT0). All parameters were computed with their 95% Confidence Limits. Statistical significance required a *P* value < 0.05. All statistical analyses were performed using SPSS statistical analysis software version 18 (Statistical Package for Social Sciences, Chicago, IL, USA).

## SUPPLEMENTARY FIGURES AND TABLES



## Abbreviations

WT: wild-type
DEN: diethylnitrosamine
HCC: hepatocellular carcinoma
*Per2*: Period 2
*Cry*: cryptochrome
mRNA: messenger RNA
CTS: circadian timing system
CCNB1: cyclin B1
Bcl2: B cell lymphoma 2
*Per2*^m/m^: *Per2* mutant mice
ZT: Zeitgeber time
qPCR: quantative polymerase chain reaction
SEM: standard error of the mean
ALAT: alanine aminotransferase
ASAT: aspartate aminotransferase
CCA: cholangiocarcinoma
