# Supplementary material for: Clock gene Per2 as a controller of liver carcinogenesis
Source: Oncotarget. 2016 Aug 3;7(52):85832–47. doi: 10.18632/oncotarget.11037 (PMC5349878; doi:10.18632/oncotarget.11037)
Supplement: Supplementary file 1 [file oncotarget-07-85832-s001.pdf]

Clock gene *Per2* as a controller of liver carcinogenesis

SUPPLEMENTARY FIGURES AND TABLES

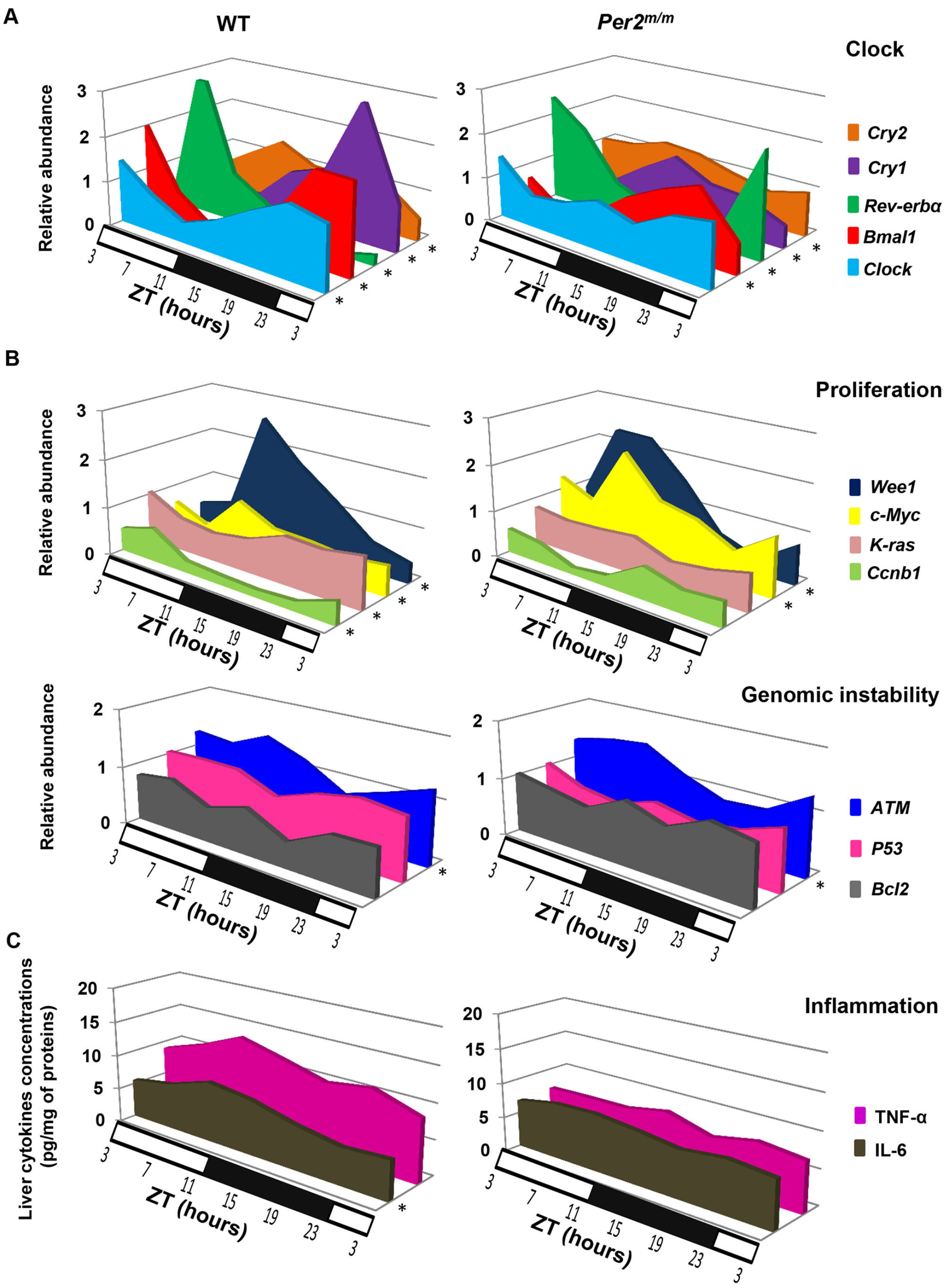

Supplementary Figure S1: Moderation of liver clock, proliferation, genomic instability and pro-inflammatory cytokine patterns by *Per2* loss-of-function, in the absence of any DEN exposure. Each data point is mean of 5 mice at each of 6 Zeitgeber Times (ZT) for WT (left panels) or *Per2*$^{m/m}$ mice (right panels). Circadian mRNA expression of **A.** clock genes *Clock, Bmal1, Rev-erbα, Cry1*, and *Cry2;* **B.** proliferation and genomic instability genes *Ccnb1, K-ras, c-Myc, Wee1, Bcl2, P53*, and *ATM*. **C.** Circadian pattern of liver cytokines IL-6 and TNF-α liver concentrations. * denotes statistical validation of 24h rhythm with Cosinor (p< 0.05).

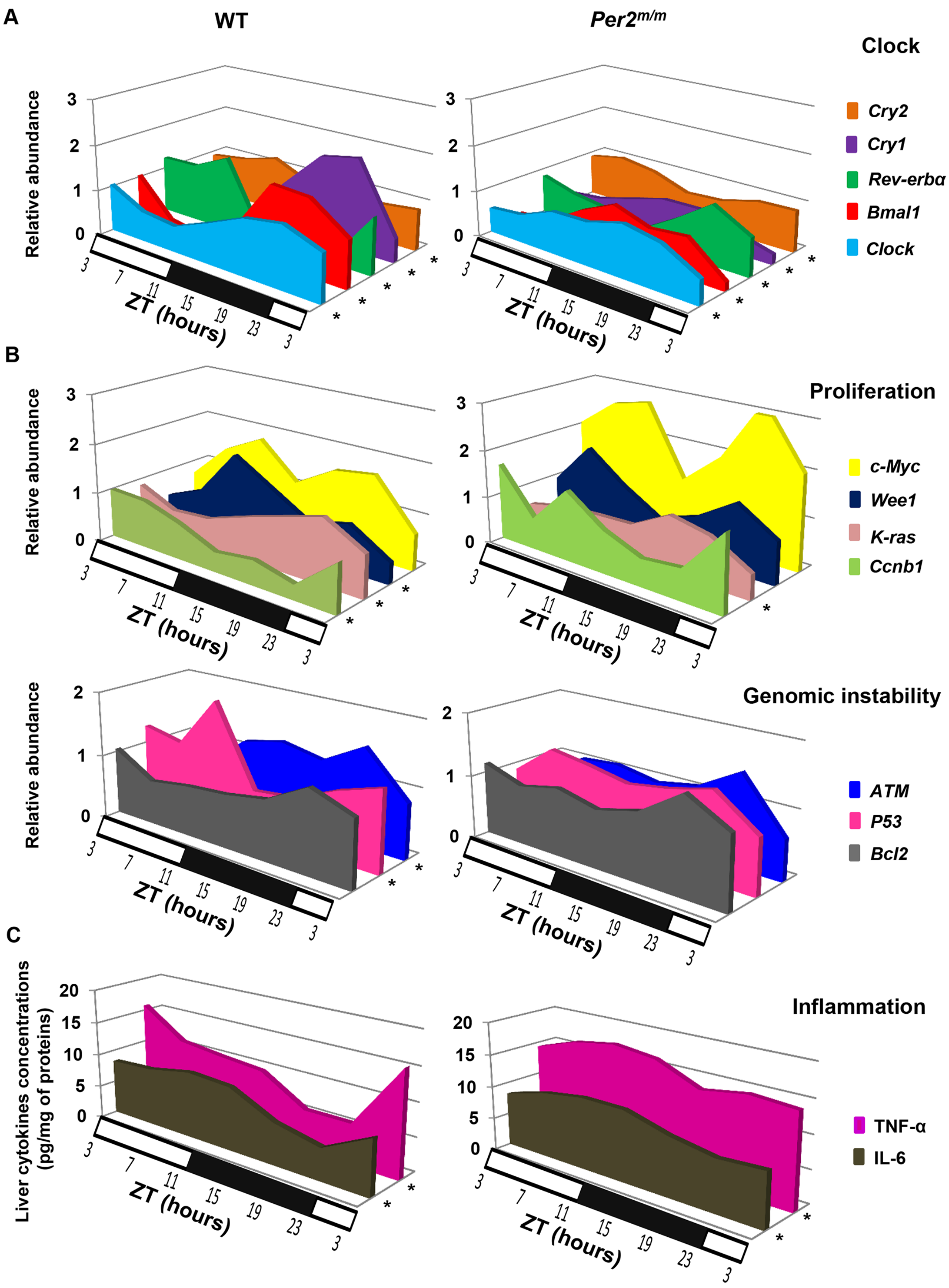

Supplementary Figure S2: DEN-induced alterations of liver circadian patterns of clock, proliferation, genomic instability and inflammation markers according to *Per2* loss-of-function during the carcinogenesis initiation stage. Each data point is mean of 5 to 7 mice at each of 6 Zeitgeber Times (ZT) for WT (left panels) or *Per2*$^{m/m}$ mice (right panels). Circadian mRNA expression of **A.** clock genes *Clock, Bmal1, Rev-erbα*, *Cry1*, and *Cry2;* **B.** proliferation and genomic instability genes *Ccnb1, K-ras, c-Myc, Wee1, Bcl2, P53*, and *ATM*. **C.** Circadian pattern of liver cytokines IL-6 and TNF-α protein concentrations. * denotes statistical validation of 24h rhythm with Cosinor ($p < 0.05$).

Supplementary Table S1: Age of mice used at the beginning of each experiment

	WT			*Per2$^{m/m}$*		
**Age (Weeks)**	**8-9**	**10-11**	**12-14**	**8-9**	**10-11**	**12-14**
Exp I	6*	12	12	6	10	14
Exp IIA			6	-	-	6
Exp IIB	9	7	14	9	14	19
Exp III	0	0	15	0	0	15

* Number of mice

Supplementary Table S2: Forward and reverse primers

Gene	Forward sequence	Reverse sequence
Clock	5'-CTTCCTGGTAACGCGAGAAAG-3'	5'-GTCGAATCTCACTAGCATCTGAC-3'
Bmal1	5'-CTCATTGATGCCAAGACTGG-3'	5'-GGTGGCCAGCTTTTCAAATA-3'
Rev-erba	5'-AACCTCCAGTTTGTGTCAAGGT-3'	5'-GATGACGATGATGCAGAAGAAG-3'
Cry1	5'-TACAGAGGGCTAGGTCTTCTCG-3'	5'-TACAGCTCGGGACGTTCTCT-3'
Cry2	5'-GCGTCTGTTTGTAGTCCGGG-3'	5'-TCCCAAAGGGTTCAGAGTCATA-3'
P53	5'-GCTTCTCCGAAGACTGGATGA-3'	5'-GACACTCGGAGGGCTTCACT-3'
ATM	5'-GATCTCGTGCCTTGGCTACA-3'	5'-CTGAGAGCTTTCCAGGTTTG-3'
Bcl2	5'-ATAACGGAGGCTGGGATGC-3'	5'-CAGGTATGCACCCAGAGTGA-3'
Wee1	5'-GAGCGTATTTTAATGATTCC-3'	5'-AGAGCCAGCCAATGGTTTT-3'
Ccnb1	5'-CTAAAGTCGGAGAGGTTGACG-3'	5'-TCCATTCACCGTTGTCAAGA-3'
C-myc	5'-TCCTGTTGGTGAAGTTCACGTT-3'	5'-CTGTTTGAAGGCTGGATTTCCT-3'
K-ras	5'-AGAACTGGGGAGGGCTTTCT-3'	5'-GCATCGTCAACACCCTGTCT-3'
